# Crystal structure of [(2*R*,3*R*,4*S*)-3,4-bis(acet­yloxy)-5-iodo-3,4-di­hydro-2*H*-pyran-2-yl]methyl acetate

**DOI:** 10.1107/S205698901402564X

**Published:** 2015-01-01

**Authors:** Julio Zukerman-Schpector, Ignez Caracelli, Hélio A. Stefani, Anwar Shamim, Edward R.T. Tiekink

**Affiliations:** a Departmento de Química, Universidade Federal de São Carlos, 13565-905 São Carlos, SP, Brazil; b Departmento de Física, Universidade Federal de São Carlos, 13565-905 São Carlos, SP, Brazil; c Departamento de Farmácia, Faculdade de Ciências Farmacêuticas, Universidade de São Paulo, São Paulo-SP, Brazil; d Instituto de Química, Universidade de São Paulo, São Paulo-SP, Brazil; e Department of Chemistry, University of Malaya, 50603 Kuala Lumpur, Malaysia

**Keywords:** Crystal structure, carbohydrate, conformation, C—H⋯O inter­actions, crystal structure

## Abstract

In the title compound, C_12_H_15_IO_7_, the 3,4-di­hydro-2*H*-pyran ring is in a distorted half-boat conformation with the atom bearing the acet­yloxy group adjacent to the C atom bearing the methyl­acetate group lying 0.633 (6) Å above the plane of the remaining ring atoms (r.m.s. deviation = 0.0907 Å). In the crystal, mol­ecules are linked into a supra­molecular chain along the *a* axis through two C—H⋯O inter­actions to the same acceptor carbonyl O atom; these chains pack with no specific inter­molecular inter­actions between them.

## Related literature   

For the structure of the unsubstituted parent compound, determined three times, and having a distorted half-boat conformation, see: Vangehr *et al.* (1979 ▸); Krajewski *et al.* (1979 ▸); Voelter *et al.* (1981 ▸).
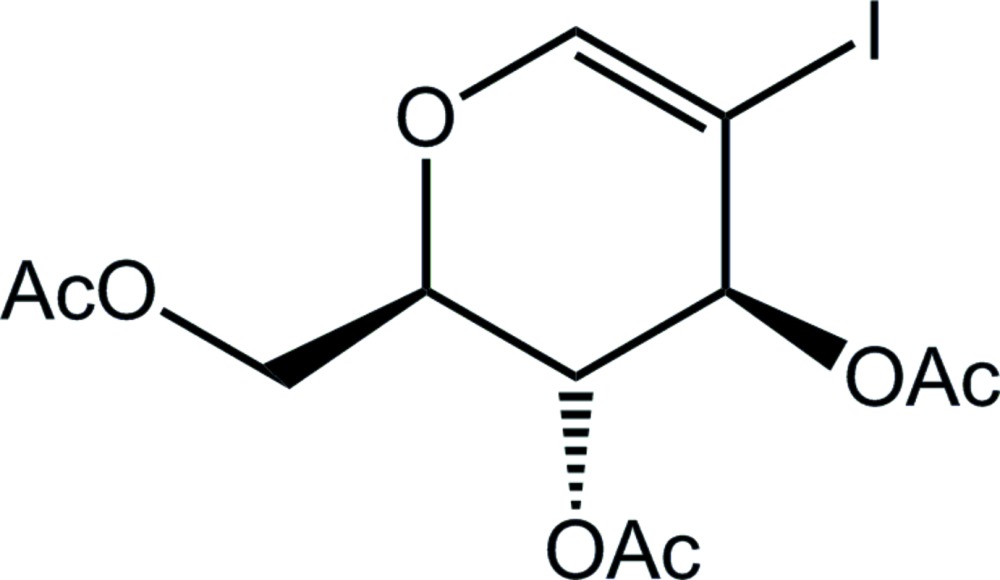


## Experimental   

### Crystal data  

C_12_H_15_IO_7_
*M* *_r_* = 398.14
Orthorhombic, 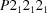

*a* = 7.9048 (2) Å
*b* = 8.7521 (2) Å
*c* = 22.7094 (5) Å
*V* = 1571.12 (6) Å^3^
*Z* = 4
Mo *K*α radiation
μ = 2.06 mm^−1^
*T* = 293 K
0.35 × 0.24 × 0.11 mm

### Data collection  

Bruker APEXII CCD diffractometer
Absorption correction: multi-scan (*SADABS*; Sheldrick, 1996 ▸) *T*_min_ = 0.601, *T*_max_ = 0.745
6116 measured reflections
2818 independent reflections
2456 reflections with *I* > 2σ(*I*)
*R*_int_ = 0.019

### Refinement  

*R*[*F* ^2^ > 2σ(*F* ^2^)] = 0.028
*wR*(*F* ^2^) = 0.071
*S* = 1.04
2818 reflections
184 parameters
H-atom parameters constrained
Δρ_max_ = 0.28 e Å^−3^
Δρ_min_ = −0.58 e Å^−3^
Absolute structure: Flack *x* determined using 925 quotients [(*I* ^+^)−(*I* ^−^)]/[(*I* ^+^)+(*I* ^−^)] (Parsons *et al.*, 2013 ▸)
Absolute structure parameter: 0.000 (11)

### 

Data collection: *APEX2* (Bruker, 2009 ▸); cell refinement: *SAINT* (Bruker, 2009 ▸); data reduction: *SAINT*; program(s) used to solve structure: *SIR* (Burla *et al.*, 2014 ▸; program(s) used to refine structure: *SHELXL2014* (Sheldrick, 2008 ▸); molecular graphics: *ORTEP-3 for Windows* (Farrugia, 2012 ▸) and *DIAMOND* (Brandenburg, 2006 ▸); software used to prepare material for publication: *MarvinSketch* (ChemAxon, 2010 ▸) and *publCIF* (Westrip, 2010 ▸).

## Supplementary Material

Crystal structure: contains datablock(s) I, New_Global_Publ_Block. DOI: 10.1107/S205698901402564X/hb7323sup1.cif

Structure factors: contains datablock(s) I. DOI: 10.1107/S205698901402564X/hb7323Isup2.hkl

Supporting information file. DOI: 10.1107/S205698901402564X/hb7323Isup3.cml

**Fig. 1.**
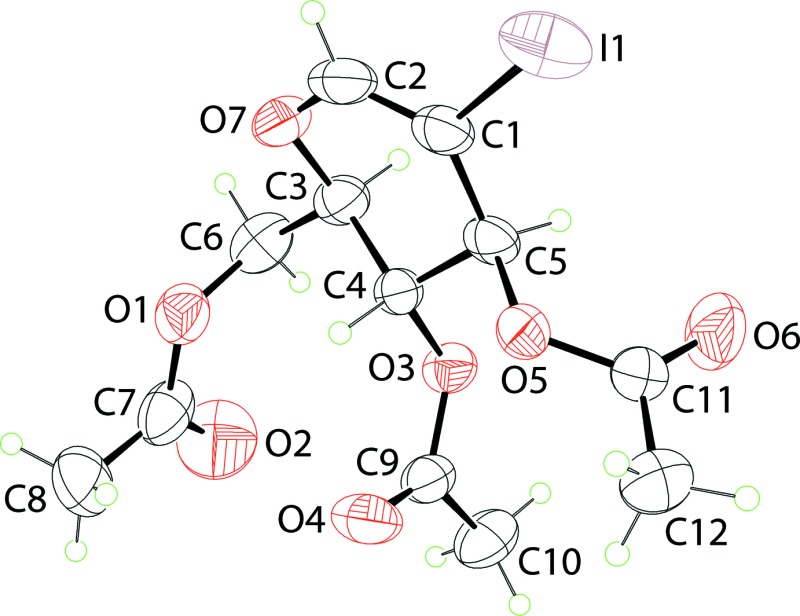
The molecular structure of the title compound showing displacement ellipsoids at the 35% probability level.

**Fig. 2.**
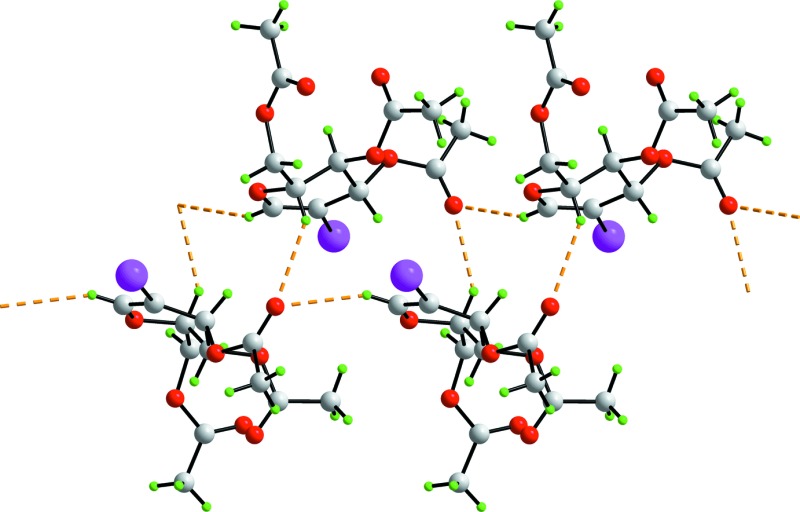
A view of the supramolecular chain along the a axis mediated by C—H···O interactions (orange dashed lines).

**Fig. 3.**
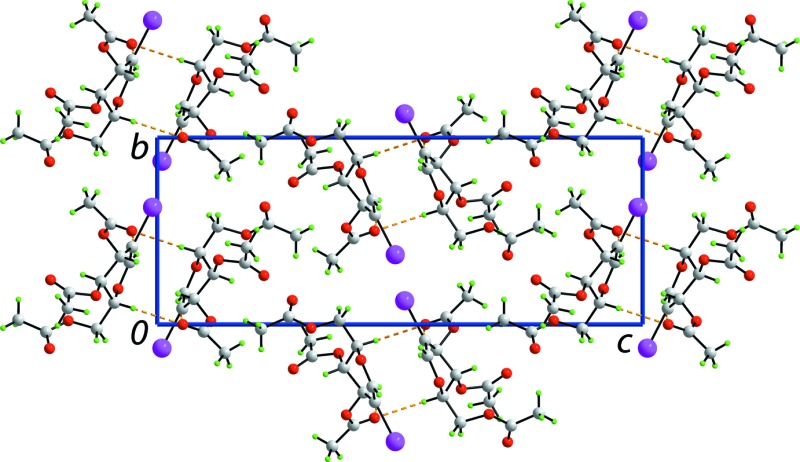
A view in projection down the a axis of the unit-cell contents. The C—H···O interactions are shown as orange dashed lines.

. DOI: 10.1107/S205698901402564X/hb7323fig1.tif
The mol­ecular structure of the title compound showing displacement ellipsoids at the 35% probability level.

a . DOI: 10.1107/S205698901402564X/hb7323fig2.tif
A view of the supra­molecular chain along the *a* axis mediated by C—H⋯O inter­actions (orange dashed lines).

a . DOI: 10.1107/S205698901402564X/hb7323fig3.tif
A view in projection down the *a* axis of the unit-cell contents. The C—H⋯O inter­actions are shown as orange dashed lines.

CCDC reference: 1035669

Additional supporting information:  crystallographic information; 3D view; checkCIF report

## Figures and Tables

**Table 1:** Hydrogen-bond geometry (, )

*D*H*A*	*D*H	H*A*	*D* *A*	*D*H*A*
C2H2O6^i^	0.93	2.58	3.448(7)	156
C3H3O6^ii^	0.98	2.55	3.383(6)	143

Symmetry codes: (i) 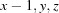; (ii) 
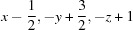
.

## supplementary crystallographic information

### S1.1. Synthesis and crystallization

To a solution of 3,4,6-tri-oxo­acetyl-D-Glucal (3 mmol) in aceto­nitrile (9 mL) at 373 K under a N_2_ atmosphere was added N-iodo­succinimide (3.6 mmol) and silver nitrate (0.6 mmol) as catalyst followed by stirring for 4 h. After consumption of the starting material (TLC monitoring), the reaction mixture was filtered through a sintered funnel (using Celite) and the filtrate was then evaporated giving a crude product which was purified by silica gel column chromatography (20-30% of EtOAc/hexane) to obtain the title compound. Suitable crystals were obtained by keeping the EtOAc solution of the product at 277 K for 48 h.

^1^H NMR (CDCl_3_, 300 MHz): δ 6.73 (s, 1H), 5.44 (d, *J* = 5.1 Hz, 1H), 5.18 (dd, *J* = 5.1, 7.0 Hz, 1>H), 4.37-4.30 (m, 2H), 4.08-4.18 (m, 1H), 2.09 (s, 3H), 2.05 (s, 3H), 2.04 (s, 3H). ^13^C NMR (CDCl_3_, 75 MHz) δ = 170.5, 170.3, 169.4, 149.4, 74.0, 70.6, 67.6, 66.3, 61.0, 20.9, 20.8, 20.7 ppm. HRMS: calcd. for C_12_H_15_IO_7_ [M + H]^+^ 397.9862; found: 397.9863.

### S1.2. Refinement

Carbon-bound H-atoms were placed in calculated positions (C—H = 0.93 to 0.98 Å) and were included in the refinement in the riding model approximation, with *U_iso_*(H) = 1.2–1.5*U_eq_*(C).

### S2. Results and discussion

The 3,4-di­hydro-2*H*-pyran ring in the title compound, Fig. 1, is in a distorted half-boat conformation as reflected in the conformational parameters: the puckering amplitude (Q) = 0.497 (5) Å, θ = 52.6 (6)° and φ = 268.6 (7)°. In this conformation, the C4 atom lies 0.633 (6) Å above the plane of the remaining ring atoms which have a r.m.s. of 0.0907 Å. The substituents at the C3 and C4 sites occupy equatorial positions while that at atom C5 is bis­ectional. The crystal structure of the unsubstituted parent compound has been reported three times and also adopts a distorted half-boat conformation (Vangehr *et al.*, 1979; Krajewski *et al.*, 1979; Voelter *et al.*, 1981).

In the crystal , the molecules are linked *via* two independent C—H···O inter­actions, Table 1, involving the same carbonyl-O6 atom as acceptor. The resulting supra­molecular architecture is a chain parallel to the *a* axis, Fig. 2. These chains pack with no specific inter­molecular inter­actions between them, Fig. 3.

### Crystal data

**Table:**

C_12_H_15_IO_7_	*D*_x_ = 1.683 Mg m^−^^3^
*M**_r_* = 398.14	Mo *K*α radiation, λ = 0.71073 Å
Orthorhombic, *P*2_1_2_1_2_1_	Cell parameters from 3256 reflections
*a* = 7.9048 (2) Å	θ = 2.7–25.1°
*b* = 8.7521 (2) Å	µ = 2.06 mm^−^^1^
*c* = 22.7094 (5) Å	*T* = 293 K
*V* = 1571.12 (6) Å^3^	Irregular, colourless
*Z* = 4	0.35 × 0.24 × 0.11 mm
*F*(000) = 784	

### Data collection

**Table:**

Bruker APEXII CCD diffractometer	2456 reflections with *I* > 2σ(*I*)
φ and ω scans	*R*_int_ = 0.019
Absorption correction: multi-scan (*SADABS*; Sheldrick, 1996)	θ_max_ = 25.4°, θ_min_ = 1.8°
*T*_min_ = 0.601, *T*_max_ = 0.745	*h* = −9→5
6116 measured reflections	*k* = −7→10
2818 independent reflections	*l* = −27→27

### Refinement

**Table:**

Refinement on *F*^2^	Secondary atom site location: difference Fourier map
Least-squares matrix: full	Hydrogen site location: inferred from neighbouring sites
*R*[*F*^2^ > 2σ(*F*^2^)] = 0.028	H-atom parameters constrained
*wR*(*F*^2^) = 0.071	*w* = 1/[σ^2^(*F*_o_^2^) + (0.0299*P*)^2^ + 0.3563*P*] where *P* = (*F*_o_^2^ + 2*F*_c_^2^)/3
*S* = 1.04	(Δ/σ)_max_ < 0.001
2818 reflections	Δρ_max_ = 0.28 e Å^−^^3^
184 parameters	Δρ_min_ = −0.58 e Å^−^^3^
0 restraints	Absolute structure: Flack *x* determined using 925 quotients [(*I*^+^)-(*I*^-^)]/[(*I*^+^)+(*I*^-^)] (Parsons *et al.*, 2013)
Primary atom site location: structure-invariant direct methods	Absolute structure parameter: 0.000 (11)

### Special details

**Table:**

Geometry. All e.s.d.'s (except the e.s.d. in the dihedral angle between two l.s. planes) are estimated using the full covariance matrix. The cell e.s.d.'s are taken into account individually in the estimation of e.s.d.'s in distances, angles and torsion angles; correlations between e.s.d.'s in cell parameters are only used when they are defined by crystal symmetry. An approximate (isotropic) treatment of cell e.s.d.'s is used for estimating e.s.d.'s involving l.s. planes.

### Fractional atomic coordinates and isotropic or equivalent isotropic displacement parameters (Å^2^)

**Table:**

	*x*	*y*	*z*	*U*_iso_*/*U*_eq_	
I	0.55529 (6)	0.37319 (5)	0.49032 (2)	0.1044 (2)	
O1	0.5090 (4)	0.9803 (5)	0.31664 (17)	0.0798 (10)	
O2	0.7080 (7)	1.1290 (7)	0.2782 (3)	0.141 (2)	
O3	0.8630 (3)	0.8456 (4)	0.37365 (13)	0.0609 (8)	
O4	0.8637 (5)	0.7615 (5)	0.28034 (15)	0.0839 (11)	
O5	0.8078 (4)	0.5163 (4)	0.38956 (12)	0.0603 (8)	
O6	1.0344 (5)	0.4998 (6)	0.44747 (19)	0.1042 (14)	
O7	0.4218 (4)	0.8098 (5)	0.41934 (15)	0.0749 (9)	
C1	0.5672 (6)	0.5851 (6)	0.44833 (17)	0.0637 (11)	
C2	0.4291 (6)	0.6670 (7)	0.44323 (19)	0.0728 (14)	
H2	0.3287	0.6246	0.4569	0.087*	
C3	0.5830 (5)	0.8834 (6)	0.41154 (19)	0.0653 (12)	
H3	0.6264	0.9136	0.4502	0.078*	
C4	0.7049 (5)	0.7701 (5)	0.38426 (17)	0.0514 (10)	
H4	0.6585	0.7334	0.3468	0.062*	
C5	0.7335 (5)	0.6361 (6)	0.42476 (16)	0.0576 (10)	
H5	0.8095	0.6647	0.4570	0.069*	
C6	0.5543 (7)	1.0241 (6)	0.3756 (3)	0.0825 (14)	
H6A	0.4643	1.0846	0.3929	0.099*	
H6B	0.6564	1.0855	0.3749	0.099*	
C7	0.5931 (7)	1.0403 (7)	0.2711 (3)	0.0887 (18)	
C8	0.5349 (9)	0.9756 (11)	0.2140 (3)	0.121 (3)	
H8A	0.5497	0.8667	0.2143	0.181*	
H8B	0.6000	1.0190	0.1824	0.181*	
H8C	0.4174	0.9993	0.2083	0.181*	
C9	0.9308 (6)	0.8302 (5)	0.3190 (2)	0.0607 (11)	
C10	1.0949 (6)	0.9124 (7)	0.3156 (3)	0.0869 (16)	
H10A	1.0799	1.0066	0.2946	0.130*	
H10B	1.1762	0.8500	0.2954	0.130*	
H10C	1.1348	0.9338	0.3547	0.130*	
C11	0.9611 (6)	0.4617 (6)	0.4043 (2)	0.0653 (11)	
C12	1.0198 (7)	0.3483 (8)	0.3605 (3)	0.0952 (19)	
H12A	1.1130	0.2915	0.3766	0.143*	
H12B	1.0556	0.4002	0.3254	0.143*	
H12C	0.9290	0.2796	0.3512	0.143*	

### Atomic displacement parameters (Å^2^)

**Table:**

	*U*^11^	*U*^22^	*U*^33^	*U*^12^	*U*^13^	*U*^23^
I	0.1334 (4)	0.0973 (3)	0.0825 (3)	−0.0195 (3)	0.0270 (2)	0.0107 (2)
O1	0.0605 (18)	0.092 (3)	0.087 (2)	−0.0091 (16)	−0.0051 (16)	0.015 (2)
O2	0.126 (4)	0.127 (4)	0.169 (5)	−0.048 (4)	0.012 (3)	0.045 (4)
O3	0.0480 (14)	0.074 (2)	0.0606 (16)	−0.0122 (14)	0.0004 (12)	−0.0116 (16)
O4	0.080 (2)	0.114 (3)	0.058 (2)	−0.022 (2)	0.0163 (18)	−0.013 (2)
O5	0.0586 (16)	0.076 (2)	0.0461 (15)	0.0052 (15)	−0.0067 (13)	−0.0102 (15)
O6	0.075 (2)	0.146 (4)	0.092 (3)	0.021 (3)	−0.030 (2)	−0.026 (3)
O7	0.0491 (16)	0.105 (3)	0.0702 (19)	0.0004 (17)	0.0113 (15)	−0.0053 (19)
C1	0.069 (3)	0.081 (3)	0.0416 (19)	−0.010 (3)	0.0058 (19)	−0.006 (2)
C2	0.066 (3)	0.102 (4)	0.050 (2)	−0.018 (3)	0.017 (2)	−0.014 (3)
C3	0.056 (2)	0.085 (3)	0.055 (2)	−0.006 (3)	0.0020 (18)	−0.020 (3)
C4	0.0427 (19)	0.069 (3)	0.042 (2)	−0.0070 (19)	−0.0024 (17)	−0.011 (2)
C5	0.059 (2)	0.077 (3)	0.0368 (18)	−0.011 (2)	−0.0018 (16)	−0.011 (2)
C6	0.068 (3)	0.073 (3)	0.107 (4)	0.001 (3)	0.008 (3)	−0.011 (3)
C7	0.063 (3)	0.086 (4)	0.118 (5)	0.007 (3)	0.004 (3)	0.041 (4)
C8	0.102 (4)	0.174 (8)	0.086 (4)	0.001 (5)	−0.008 (4)	0.057 (5)
C9	0.052 (2)	0.065 (3)	0.065 (3)	0.002 (2)	0.010 (2)	0.002 (2)
C10	0.059 (3)	0.092 (4)	0.109 (4)	−0.010 (3)	0.017 (3)	0.007 (3)
C11	0.058 (2)	0.084 (3)	0.055 (2)	0.001 (3)	0.003 (2)	0.005 (2)
C12	0.086 (4)	0.116 (5)	0.084 (4)	0.023 (4)	0.010 (3)	−0.013 (4)

### Geometric parameters (Å, º)

**Table:**

I—C1	2.087 (5)	C4—C5	1.507 (6)
O1—C7	1.336 (7)	C4—H4	0.9800
O1—C6	1.438 (7)	C5—H5	0.9800
O2—C7	1.205 (8)	C6—H6A	0.9700
O3—C9	1.359 (5)	C6—H6B	0.9700
O3—C4	1.434 (5)	C7—C8	1.490 (10)
O4—C9	1.188 (6)	C8—H8A	0.9600
O5—C11	1.345 (6)	C8—H8B	0.9600
O5—C5	1.444 (5)	C8—H8C	0.9600
O6—C11	1.186 (6)	C9—C10	1.486 (7)
O7—C2	1.364 (7)	C10—H10A	0.9600
O7—C3	1.439 (5)	C10—H10B	0.9600
C1—C2	1.311 (7)	C10—H10C	0.9600
C1—C5	1.488 (6)	C11—C12	1.479 (7)
C2—H2	0.9300	C12—H12A	0.9600
C3—C6	1.495 (8)	C12—H12B	0.9600
C3—C4	1.515 (6)	C12—H12C	0.9600
C3—H3	0.9800		
			
C7—O1—C6	119.4 (5)	O1—C6—H6B	109.9
C9—O3—C4	116.8 (3)	C3—C6—H6B	109.9
C11—O5—C5	119.1 (3)	H6A—C6—H6B	108.3
C2—O7—C3	114.9 (4)	O2—C7—O1	121.7 (7)
C2—C1—C5	122.6 (5)	O2—C7—C8	126.4 (6)
C2—C1—I	119.3 (4)	O1—C7—C8	111.8 (5)
C5—C1—I	118.1 (4)	C7—C8—H8A	109.5
C1—C2—O7	124.9 (4)	C7—C8—H8B	109.5
C1—C2—H2	117.6	H8A—C8—H8B	109.5
O7—C2—H2	117.6	C7—C8—H8C	109.5
O7—C3—C6	107.6 (4)	H8A—C8—H8C	109.5
O7—C3—C4	108.7 (4)	H8B—C8—H8C	109.5
C6—C3—C4	114.3 (4)	O4—C9—O3	123.3 (4)
O7—C3—H3	108.7	O4—C9—C10	126.6 (4)
C6—C3—H3	108.7	O3—C9—C10	110.1 (4)
C4—C3—H3	108.7	C9—C10—H10A	109.5
O3—C4—C5	109.3 (3)	C9—C10—H10B	109.5
O3—C4—C3	108.7 (3)	H10A—C10—H10B	109.5
C5—C4—C3	110.8 (4)	C9—C10—H10C	109.5
O3—C4—H4	109.3	H10A—C10—H10C	109.5
C5—C4—H4	109.3	H10B—C10—H10C	109.5
C3—C4—H4	109.3	O6—C11—O5	123.1 (5)
O5—C5—C1	109.9 (4)	O6—C11—C12	126.2 (5)
O5—C5—C4	106.8 (3)	O5—C11—C12	110.7 (4)
C1—C5—C4	108.7 (4)	C11—C12—H12A	109.5
O5—C5—H5	110.5	C11—C12—H12B	109.5
C1—C5—H5	110.5	H12A—C12—H12B	109.5
C4—C5—H5	110.5	C11—C12—H12C	109.5
O1—C6—C3	109.1 (4)	H12A—C12—H12C	109.5
O1—C6—H6A	109.9	H12B—C12—H12C	109.5
C3—C6—H6A	109.9		
			
C5—C1—C2—O7	−3.9 (7)	C2—C1—C5—C4	−13.0 (6)
I—C1—C2—O7	176.7 (3)	I—C1—C5—C4	166.4 (3)
C3—O7—C2—C1	−13.7 (6)	O3—C4—C5—O5	−76.7 (4)
C2—O7—C3—C6	170.0 (4)	C3—C4—C5—O5	163.5 (3)
C2—O7—C3—C4	45.7 (5)	O3—C4—C5—C1	164.8 (3)
C9—O3—C4—C5	108.8 (4)	C3—C4—C5—C1	45.0 (4)
C9—O3—C4—C3	−130.1 (4)	C7—O1—C6—C3	−128.3 (5)
O7—C3—C4—O3	177.0 (3)	O7—C3—C6—O1	−68.9 (5)
C6—C3—C4—O3	56.8 (5)	C4—C3—C6—O1	51.9 (5)
O7—C3—C4—C5	−62.8 (4)	C6—O1—C7—O2	1.0 (8)
C6—C3—C4—C5	177.0 (4)	C6—O1—C7—C8	177.0 (5)
C11—O5—C5—C1	−121.9 (4)	C4—O3—C9—O4	1.9 (6)
C11—O5—C5—C4	120.4 (4)	C4—O3—C9—C10	−179.0 (4)
C2—C1—C5—O5	−129.5 (5)	C5—O5—C11—O6	5.3 (7)
I—C1—C5—O5	49.9 (4)	C5—O5—C11—C12	−175.9 (4)

### Hydrogen-bond geometry (Å, º)

**Table:**

*D*—H···*A*	*D*—H	H···*A*	*D*···*A*	*D*—H···*A*
C2—H2···O6^i^	0.93	2.58	3.448 (7)	156
C3—H3···O6^ii^	0.98	2.55	3.383 (6)	143

Symmetry codes: (i) *x*−1, *y*, *z*; (ii) *x*−1/2, −*y*+3/2, −*z*+1.
