# Supplementary figures and images for: Crystal structure of [(2R,3R,4S)-3,4-bis(acet­yloxy)-5-iodo-3,4-di­hydro-2H-pyran-2-yl]methyl acetate
Source: Acta Crystallogr E Crystallogr Commun. 2015 Jan 1;71(Pt 1):o53–4. doi: 10.1107/S205698901402564X (PMC4331918; doi:10.1107/S205698901402564X)

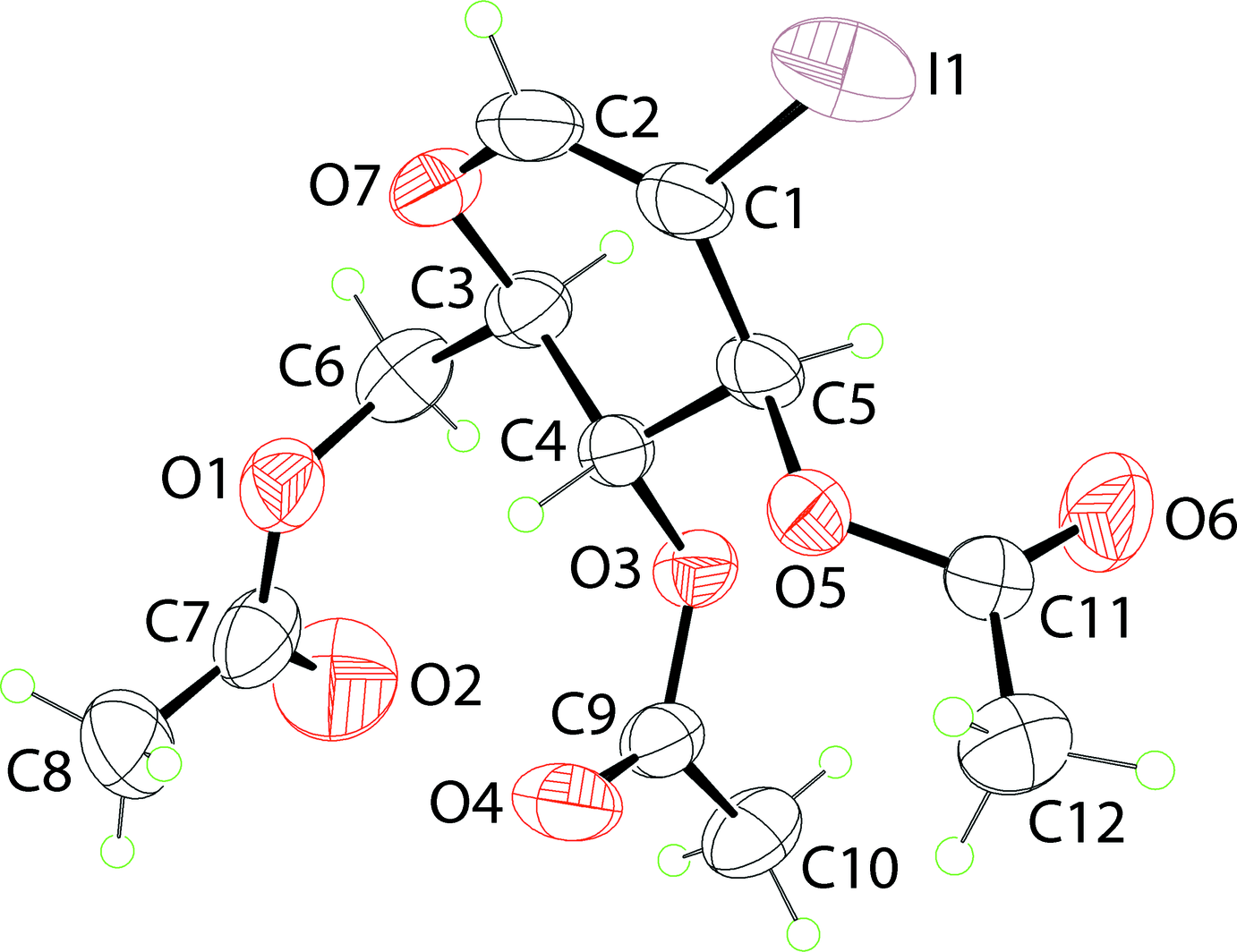

Supplement: Supplementary file 4 [file e-71-00o53-fig1.tif]

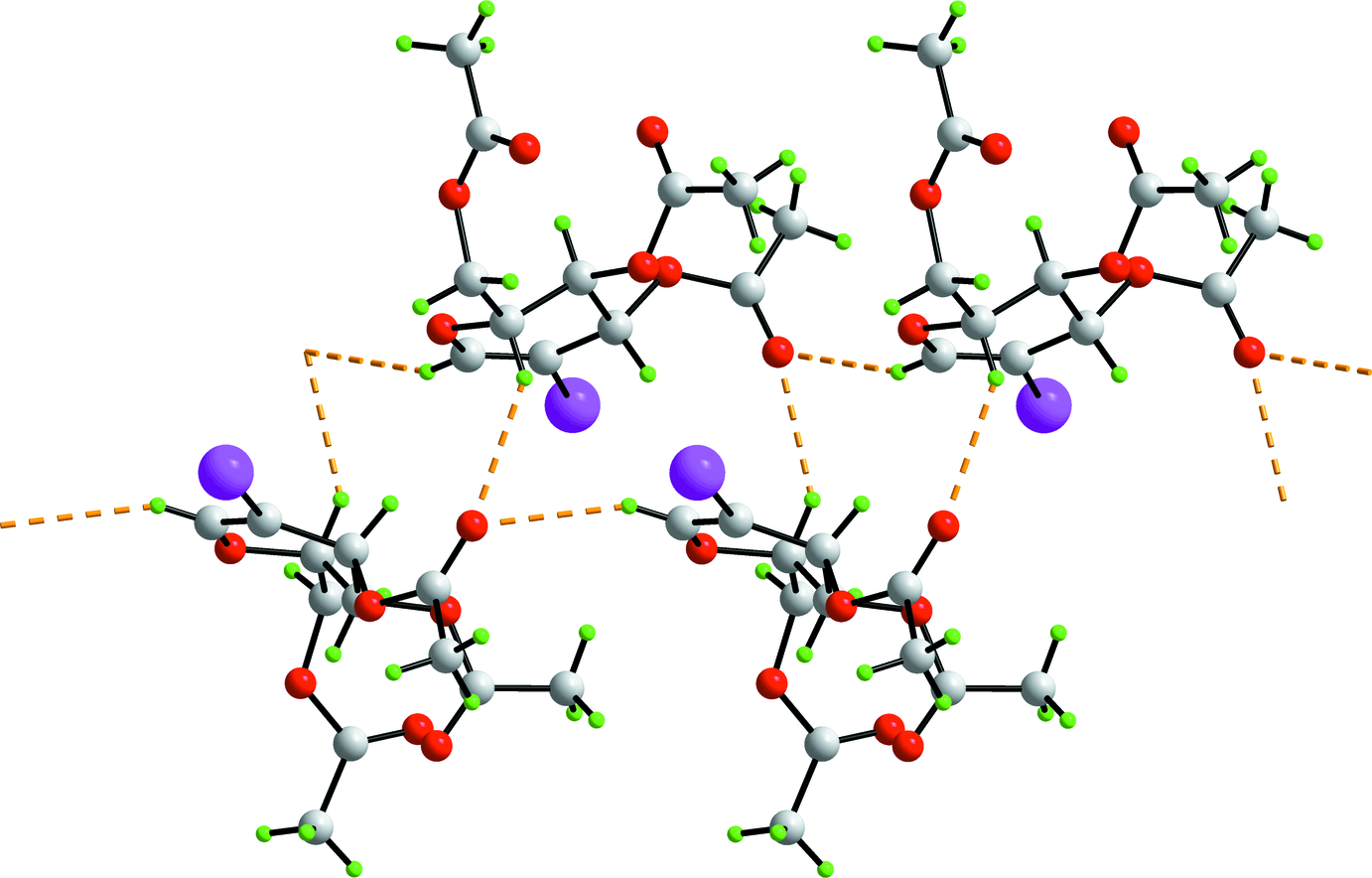

Supplement: Supplementary file 5 [file e-71-00o53-fig2.tif]

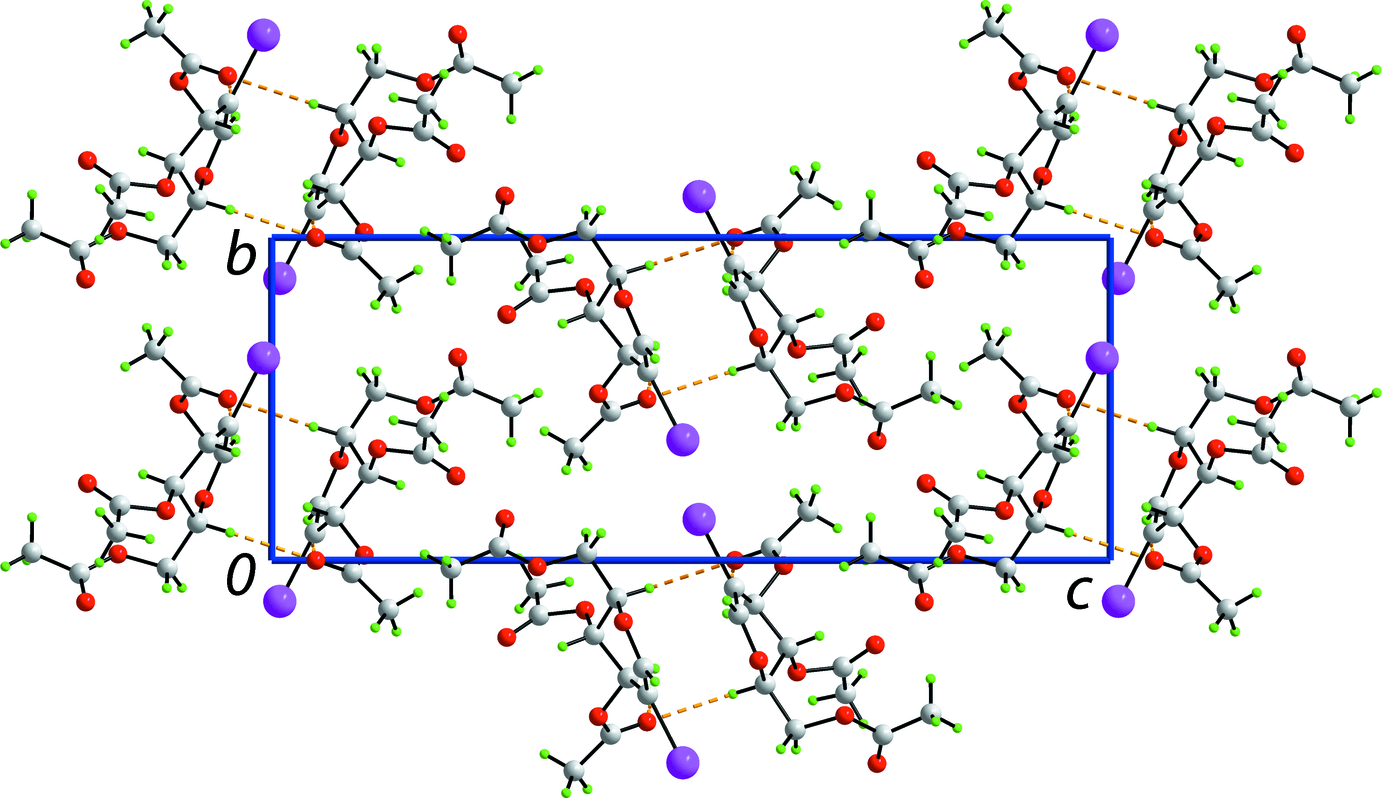

Supplement: Supplementary file 6 [file e-71-00o53-fig3.tif]
